# Overcoming immunotherapy resistance in glioblastoma: challenges and emerging strategies

**DOI:** 10.3389/fphar.2025.1584688

**Published:** 2025-03-28

**Authors:** Maowu Fu, Bing Xue, Xiuming Miao, Zong Gao

**Affiliations:** ^1^ Department of Neurosurgery, Affiliated Hospital of Shandong University of Traditional Chinese Medicine, Jinan, Shandong, China; ^2^ Department of Neurosurgery, Jinan Third People’s Hospital, Jinan, Shandong, China; ^3^ Department of Pathology, Affiliated Hospital of Shandong University of Traditional Chinese Medicine, Jinan, Shandong, China

**Keywords:** glioblastoma, immunotherapy resistance, cell therapy, TME, CAR-T

## Abstract

Glioblastoma (GBM) is the most common and aggressive primary brain tumor in adults, characterized by rapid proliferation, extensive infiltration, and significant intratumoral heterogeneity. Despite advancements in conventional treatments, including surgery, radiotherapy, and chemotherapy, the prognosis for GBM patients remains poor, with a median survival of approximately 15 months. Immunotherapy has emerged as a promising alternative; however, the unique biological and immunological features, including its immunosuppressive tumor microenvironment (TME) and low mutational burden, render it resistant to many immunotherapeutic strategies. This review explores the key challenges in GBM immunotherapy, focusing on immune evasion mechanisms, the blood-brain barrier (BBB), and the TME. Immune checkpoint inhibitors and CAR-T cells have shown promise in preclinical models but have limited clinical success due to antigen heterogeneity, immune cell exhaustion, and impaired trafficking across the BBB. Emerging strategies, including dual-targeting CAR-T cells, engineered immune cells secreting therapeutic molecules, and advanced delivery systems to overcome the BBB, show potential for enhancing treatment efficacy. Addressing these challenges is crucial for improving GBM immunotherapy outcomes.

## 1 Introduction

Glioblastoma (GBM), the most common and aggressive primary brain tumor in adults, represents a formidable challenge in oncology (Weller et al., 2024; Schaff and Mellinghoff, 2023). It is characterized by rapid proliferation, extensive infiltration into surrounding brain tissue, and significant intratumoral heterogeneity (Venkataramani et al., 2022). Despite advances in conventional treatments, including maximal safe surgical resection, radiotherapy, and temozolomide chemotherapy, the median survival for GBM patients remains approximately 15 months (Liu et al., 2023a). This grim prognosis has driven the exploration of immunotherapy as a promising alternative. However, GBM’s unique biological and immunological features render it resistant to many immunotherapeutic approaches (Liu et al., 2023a; Rong et al., 2022).

GBM is often referred to as an immunologically “cold” tumor due to its poor immunogenicity and highly suppressive tumor microenvironment (TME) (Mondal et al., 2023; Pang et al., 2023). Unlike cancers with a high mutational burden that generate numerous neoantigens capable of triggering robust immune responses, GBM has a relatively moderate mutational burden, which limits the activation of tumor-specific T cells. Furthermore, GBM cells actively downregulate major histocompatibility complex (MHC) molecules, further impairing antigen presentation and immune recognition (Ma et al., 2022). The blood-brain barrier (BBB), once considered to render the central nervous system (CNS) immune-privileged, introduces an additional challenge by limiting the infiltration of immune cells and therapeutic agents into the tumor site.

Immunotherapy has transformed the treatment landscape for several cancers (Liu et al., 2023b), yet its success in GBM has been limited. Immune checkpoint inhibitors (ICIs), such as those targeting programmed death-1 (PD-1), programmed death ligand-1 (PD-L1), and cytotoxic T lymphocyte-associated antigen-4 (CTLA-4), have shown remarkable efficacy in malignancies like melanoma and non-small cell lung cancer. Unfortunately, clinical trials in GBM have yielded disappointing results, with minimal improvements in survival (Liu et al., 2024a). This is partly due to the lack of pre-existing T cell infiltration in GBM tumors, a prerequisite for ICIs to exert their effects. Adoptive cell therapies (ACTs), including chimeric antigen receptor (CAR) T cells, have demonstrated promise in preclinical models of GBM. CAR-T cells engineered to target specific GBM antigens, such as epidermal growth factor receptor variant III (EGFRvIII) and interleukin-13 receptor alpha 2 (IL13Rα2), have shown potent anti-tumor activity *in vitro* and *in vivo* (Hoogstrate et al., 2022; Bagley et al., 2024a). However, their clinical application has been hindered by antigen heterogeneity, limited CAR-T cell persistence, and poor trafficking across the BBB. Other ACTs, such as CAR-natural killer (NK) cells and tumor-infiltrating lymphocytes (TILs), are being explored to address these limitations, but their efficacy in GBM remains to be fully elucidated. In addition, the GBM TME is a significant barrier to the success of immunotherapy (Zirem et al., 2024; Kirschenbaum et al., 2024). It is dominated by immunosuppressive cells, including tumor-associated macrophages (TAMs), myeloid-derived suppressor cells (MDSCs), and regulatory T cells (Tregs) (Bikfalvi et al., 2023). TAMs, which constitute up to 50% of the tumor mass, adopt an anti-inflammatory phenotype, secreting cytokines such as interleukin-10 (IL-10) and transforming growth factor-beta (TGF-β) that inhibit effector T cell activity (Kloosterman et al., 2024; Wang S. et al., 2023). MDSCs further suppress T cell proliferation and function, while Tregs curtail immune responses through direct cell-cell interactions and the release of inhibitory cytokines (Lin et al., 2024).

Metabolic factors also play a critical role in immunotherapy resistance. GBM is a highly glycolytic tumor, producing large amounts of lactate that acidify the TME (Guo et al., 2022; Wang et al., 2021; Wang R. et al., 2024). This metabolic shift not only promotes tumor growth but also impairs the function of infiltrating immune cells. Finally, the BBB remains a formidable obstacle to effective immunotherapy (Ricklefs et al., 2024). In this review, we will discuss all different mechanisms suppress the immunotherapy and discuss strategies to overcome these physical and biochemical barriers for improving the efficacy of immunotherapy in GBM.

## 2 Key mechanisms of immuno-resistance in glioblastoma

### 2.1 Tumor heterogeneity contributes to immunotherapy resistance in GBM

**FIGURE 1:**
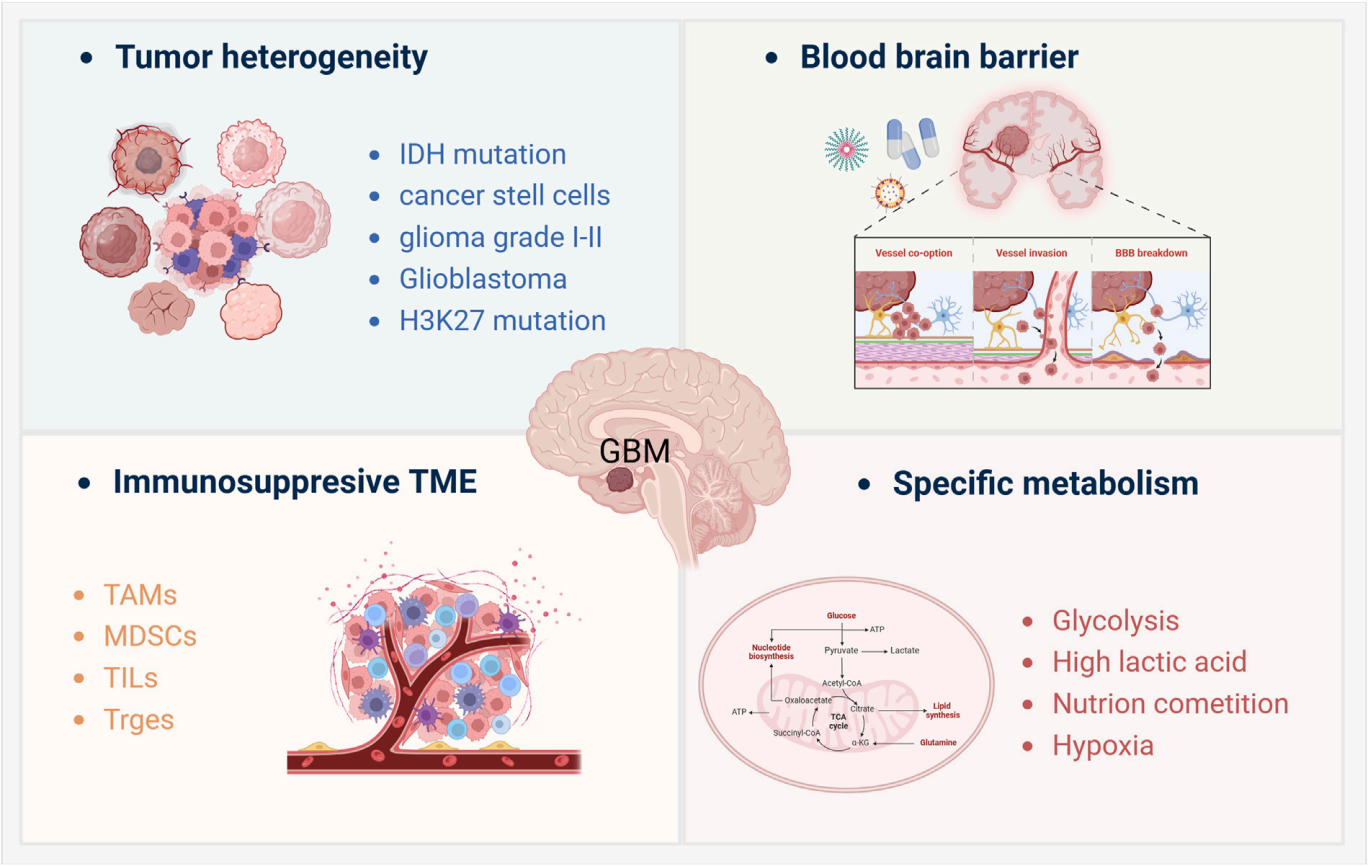
Key factors modulate the immunotherapy resistance of GBM treatment. This schematic illustrates four major factors contributing to glioblastoma (GBM) pathophysiology and immunotherapy resistance: (Weller et al., 2024): Tumor heterogeneity, characterized by diverse genetic and epigenetic alterations such as *IDH mutation, H3K27 mutation*, and the presence of *cancer stem cells*, leading to varied tumor subtypes from low-grade gliomas (Grade I-II) to glioblastoma; (Schaff and Mellinghoff, 2023); Blood-brain barrier (BBB) dysfunction, including mechanisms like *vascular co-option, vessel invasion, and BBB breakdown*, which limit drug delivery and immune cell infiltration; (Venkataramani et al., 2022); Immunosuppressive tumor microenvironment (TME), dominated by *tumor-associated macrophages (TAMs), myeloid-derived suppressor cells (MDSCs),* and regulatory immune cells such as *Tregs* and *tumor-infiltrating lymphocytes (TILs),* which collectively dampen anti-tumor immune responses; (Liu et al., 2023a); Specific metabolic adaptations, including enhanced *glycolysis, high lactic acid production, nutrient competition*, and *tumor-associated hypoxia*, all of which contribute to immune evasion and therapeutic resistance. These interconnected factors highlight the challenges in developing effective immunotherapies for GBM.

Tumor heterogeneity is one of the hallmark features of GBM and a primary driver of its resistance to immunotherapy. GBM exhibits both interpatient and intratumoral heterogeneity, characterized by diverse genetic, epigenetic, and phenotypic profiles among tumor cells. Molecularly, GBM exhibits distinct subtypes based on key genetic alterations, such as IDH mutations, H3K27 alterations (e.g., H3K27me3 loss due to EZH2 dysfunction), as well as mutations in H3F3A leading to H3K27M oncogenic transformation (Sturm et al., 2012), EGFR amplification, and TERT promoter mutations, each contributing to diverse tumor behaviors and therapeutic responses (Chai et al., 2024; Hadad et al., 2023). This diversity enables subpopulations of tumor cells to evade targeted therapies and immune responses, fostering resistance and recurrence (Mathur et al., 2024; Wu et al., 2023). Recent advancements in single-cell RNA sequencing (scRNA-seq) and spatial transcriptomics have provided deeper insights into GBM heterogeneity at the single-cell level, revealing distinct immune evasion mechanisms within different tumor subpopulations. These technologies enable the identification of immunosuppressive niches and the characterization of tumor-infiltrating immune cells, paving the way for more precise immunotherapy strategies. For example, scRNA-seq studies have uncovered unique transcriptomic profiles of glioma-associated macrophages that contribute to T-cell dysfunction, suggesting novel targets for therapeutic intervention (Ochocka et al., 2021). Additionally, GBM displays significant intratumoral heterogeneity, with coexisting populations of tumor cells exhibiting differential expression of immune checkpoints, antigen presentation machinery, and resistance mechanisms, thereby complicating the development of targeted immunotherapies. For example, epidermal growth factor receptor (EGFR) is amplified in approximately 50% of GBM cases (Brennan et al., 2013), its mutant variant EGFRvIII is expressed only in a subset of these tumors. The expression ratios of EGFRvIII to EGFR vary significantly among tumors, ranging from 1% to 95% (Hoogstrate et al., 2022). Meanwhile, the shorter C-terminal subunit (MUC1-C) is upregulated in EGFRvIII-positive glioblastoma cells, promoting tumor progression and TMZ resistance by stabilizing EGFRvIII. Its knockdown increases EGFRvIII lysosomal degradation, reducing cell survival (Tong et al., 2023). CAR-T cells targeting EGFRvIII have shown promise in preclinical models but face challenges in clinical settings because of antigen loss or heterogeneous expression. Recent findings from a phase 1 clinical trial indicate that repeated peripheral infusions of anti-EGFRvIII CAR T cells in combination with pembrolizumab show no efficacy in glioblastoma (Bagley et al., 2024a) (Figure 1).

### 2.2 Blood-brain barrier restricts immunotherapy efficacy in GBM

The BBB serves as a critical defense mechanism for the CNS, but it also poses a significant obstacle to immunotherapy. The BBB is composed of endothelial cells joined by tight junctions, astrocytic end-feet, and pericytes, forming a highly selective barrier that limits the penetration of large molecules, including therapeutic agents and immune cells (van Tellingen et al., 2015). The integrity of the BBB is variably disrupted. While some regions of the tumor exhibit a leaky BBB, allowing limited drug and immune cell infiltration, other regions remain intact and inaccessible. This heterogeneity complicates the consistent delivery of therapeutic agents. For instance, immune checkpoint inhibitors such as anti-PD-1 and anti-CTLA-4 antibodies show limited efficacy in GBM, partly because they fail to accumulate in sufficient concentrations at the tumor site due to the BBB (Pol et al., 2024).

### 2.3 The immunosuppressive TME hinders the immunotherapy efficacy in GBM

The immunosuppressive TME of GBM is a significant contributor to therapy resistance, characterized by a high infiltration of Tregs, MDSCs, and TAMs. These cells create a hostile environment for effector immune cells, suppressing anti-tumor immunity and promoting tumor growth.

#### 2.3.1 Regulatory T cells

Tregs play a pivotal role in maintaining immune homeostasis but are co-opted by GBM to suppress anti-tumor immune responses. Tregs accumulate in the TME through the secretion of chemokines such as CCL22 by tumor cells and TAMs (Azambuja et al., 2020). Once recruited, Tregs inhibit CTL activity through the release of inhibitory cytokines like TGF-β and IL-10 and the expression of immune checkpoints (Lin et al., 2024). In glioblastoma, Tregs suppress CD8^+^ T cell activation, which limits the efficacy of immune checkpoint blockade (α-PD-1) therapy. While radiotherapy enhances T cell infiltration, it also promotes the accumulation of CD103^+^ Tregs with upregulated lipid metabolism, which promotes Treg stability and survival and further reinforces immunosuppression. This metabolic shift is facilitated by the upregulation of enzymes such as acetyl-CoA carboxylase (ACC), which fuels the synthesis of fatty acids essential for Treg membrane structure and function (Kao et al., 2023). These Tregs also enhance their ability to block T cell priming and activation through lipid-based signals, such as the secretion of prostaglandins, further reinforcing immunosuppression. Recent studies have shown that in patients with GBM, high levels of Tregs are associated with poor prognosis and reduced response to therapies, including radiotherapy and immune checkpoint blockade (ICB) (Wang X. et al., 2024). Targeting Tregs facilitates tertiary lymphoid structure (TLS) formation, boosts CD4^+^ and CD8^+^ T cell function, and enhances the efficacy of radio-immunotherapy, highlighting a key mechanism of resistance and a potential therapeutic target in glioblastoma (van Hooren et al., 2023).

#### 2.3.2 Myeloid-derived suppressor cells

In GBM, MDSCs are key immunosuppressive regulators within the TME. Glioma cells reprogram metabolism to shape the TME, suppressing anti-tumor immune responses by impairing T cells, NK cells, and dendritic cells (DCs) while promoting angiogenesis and tumor progression. MDSCs, along with glioma-associated microglia/macrophages (GAMMs), are the most abundant myeloid cells in glioblastoma, driving immune evasion (Won et al., 2019; Lasser et al., 2024). MDSCs suppress T cell proliferation and function through multiple mechanisms, including the production of arginase-1 and inducible nitric oxide synthase (iNOS), which deplete essential nutrients required for T cell activity (Hegde et al., 2021). MDSCs also secrete reactive oxygen species (ROS) and TGF-β, further impairing effector T cell responses. In GBM, MDSCs contribute to radiation-induced lymphopenia, exacerbating immunosuppression and worsening patient survival. GBM patients has higher MDSC regulatory genes and increased circulating MDSCs in lymphopenic patients post-chemoradiotherapy. Preclinical models confirmed that MDSCs drive systemic lymphopenia, and their depletion improved survival. Pharmacological inhibition of MDSCs using arginase-1 inhibitor (CB1158) or PDE-5 inhibitor (tadalafil) successfully mitigated radiation-induced lymphopenia, highlighting MDSCs as a key therapeutic target to enhance anti-tumor immunity in glioblastoma (Ghosh et al., 2023).

#### 2.3.3 Tumor-associated macrophages

In glioblastoma, TAMs play a crucial role in promoting tumor progression through metabolic and signaling interactions with tumor cells. Glioblastoma cells enhance TAM recruitment by activating the LDHA-ERK-YAP1/STAT3 axis, leading to CCL2 and CCL7 secretion, which attracts macrophages into the TME. In turn, TAMs secrete LDHA-containing extracellular vesicles, fueling glioblastoma glycolysis, proliferation, and survival, establishing a tumor-macrophage symbiosis (Khan et al., 2024). TAMs represent up to 50% of the GBM tumor mass and are predominantly polarized toward an anti-inflammatory, pro-tumorigenic M2 phenotype. M2-like TAMs produce immunosuppressive cytokines (e.g., IL-10, TGF-β) and promote tissue remodeling and angiogenesis, supporting tumor progression (Blitz et al., 2022). Reprogramming TAMs toward a pro-inflammatory M1 phenotype have shown promise in preclinical studies (Liu et al., 2024b). For example, CD47-SIRPα blockade, which enhances macrophage-mediated phagocytosis of tumor cells, is being explored as a potential therapeutic approach in GBM (Zhang P. et al., 2023).

### 2.4 GBM metabolism contributes to immune resistance in GBM

Metabolic adaptations of GBM play a crucial role in shaping its immunosuppressive environment. The tumor’s reliance on aerobic glycolysis, or the Warburg effect, leads to the accumulation of lactate and acidification of the TME. High lactate levels suppress T cell and NK cell function while promoting the immunosuppressive activity of Tregs and MDSCs (Khan et al., 2024). Hypoxia, another hallmark of GBM, exacerbates immune resistance by upregulating hypoxia-inducible factors (HIFs). HIF-1α drives the expression of VEGF, promoting angiogenesis and creating physical barriers to immune cell infiltration (Domenech et al., 2021). Hypoxia also induces the expression of PD-L1 on tumor cells, further dampening T cell responses (Michelucci et al., 2023). Hypoxia in glioblastoma enhances immunosuppression by upregulating HIF1α, which drives legumain (LGMN) expression in TAMs. LGMN promotes TAM immunosuppressive polarization via the GSK-3β-STAT3 pathway, weakening anti-tumor immunity. Targeting HIF1α-LGMN signaling reduces immunosuppression and enhances anti-PD1 therapy, offering a potential therapeutic strategy for GBM(7). The activated HIF signallying in hypoxia also drives metabolic and epigenetic changes that enhance tumor heterogeneity. It also regulates Hippo-YAP/TAZ signaling, influencing downstream effectors and metabolic adaptation, making the hypoxia–YAP/TAZ axis a potential therapeutic target (Castillo et al., 2024). Additionally, GBM cells compete with immune cells for critical nutrients such as glucose and glutamine. By depleting these resources, GBM starves effector immune cells, impairing their proliferation and function. Dual targeting of glutamine metabolism and lysosomal lipid metabolism effectively inhibits glioblastoma progression (Zhong et al., 2024). For example, the upregulation of indoleamine 2,3-dioxygenase (IDO) by GBM cells catabolizes tryptophan, an amino acid essential for T cell activity, into immunosuppressive metabolites such as kynurenine (Hoogstrate et al., 2022).

## 3 Advancements in cell therapeutics for GBM

Recent developments in adaptive cellular therapeutics have shown promise in addressing the challenges posed by glioblastoma. This section explores two key areas: CAR-T cell therapy and engineered immune cells secreting therapeutic molecules.

### 3.1 CAR-T cell therapy: progress in targeting GBM-Specific antigens

CAR-T cell therapy involves modifying a patient’s T cells to express chimeric antigen receptors that recognize specific tumor antigens. In GBM, two notable targets have been identified.

#### 3.1.1 CAR-T targeting EGFRvIII

A mutant form of EGFR found in approximately 30% of GBM cases. EGFRvIII is a tumor-specific mutation that promotes oncogenic signaling (An et al., 2018). CAR-T cells targeting EGFRvIII have been developed to exploit this specificity (O'Rourke et al., 2017). However, clinical outcomes have been mixed due to antigen heterogeneity and the tumor’s ability to downregulate EGFRvIII expression, leading to therapeutic resistance. For instance, a study demonstrated that while EGFRvIII-targeted CAR-T cells could initially reduce tumor burden, the emergence of EGFRvIII-negative tumor cells resulted in disease progression (Bagley et al., 2024a).

#### 3.1.2 CAR-T targeting Interleukin-13 receptor alpha 2 (IL13Rα2)

Overexpressed in more than 75% of GBM tumors. IL13Rα2 is another attractive target due to its limited expression in normal tissues and high prevalence in GBM. CAR-T cells directed against IL13Rα2 have shown encouraging results. In a clinical trial (NCT02208362), a patient with recurrent multifocal glioblastoma received IL13Rα2-targeted CAR T cells via intracranial infusions over 220 days. Treatment was well tolerated with no grade ≥3 toxicity and led to complete regression of intracranial and spinal tumors, accompanied by increased cytokines and immune cells in cerebrospinal fluid. The response lasted 7.5 months post-treatment (Brown et al., 2016). An allogeneic, IL13Rα2-targeted CAR T cell product was developed to overcome the limitations of autologous CAR T therapy for GBM. A phase 1 trial demonstrated its feasibility, safety, and potential efficacy, with transient tumor reduction observed in some patients, supporting further investigation (Brown et al., 2022). Another pilot trial demonstrated the feasibility and safety of intracranial delivery of IL13Rα2-targeted CAR T cells in three patients with recurrent GBM. The treatment was well-tolerated, with transient anti-glioma responses observed in two patients, supporting further development of CAR T-cell therapy for GBM (Brown et al., 2015).

Enhancing the efficacy of CAR-T cells can also be achieved by engineering them to secrete cytokines that promote a pro-inflammatory TME. For example, CAR-T cells have been designed to release interleukin-12 (IL-12), a cytokine that stimulates immune responses and counteracts immunosuppressive elements within the TME (54). Preclinical models have shown that IL-12-secreting CAR-T cells exhibit improved anti-tumor activity against GBM, suggesting a promising avenue for therapy development (Agliardi et al., 2021).

### 3.2 Dual/tri-targeting strategies of CAR-T cell therapy in GBM treatment

To overcome tumor heterogeneity and antigen escape, researchers have developed CAR-T cells that simultaneously target both EGFRvIII and IL13Rα2. A phase 1 trial tested intrathecal CAR T cells targeting EGFR and IL13Rα2 in six recurrent GBM patients (NCT05168423) (Bagley et al., 2024b). Treatment showed preliminary safety and bioactivity, with early neurotoxicity managed clinically. Tumor reduction was observed, but no objective responses met criteria. Further studies are needed to confirm efficacy (Bagley et al., 2024b). In another study a bispecific IL-13Rα2/TGF-β CAR-T cell was developed to overcome the immunosuppressive GBM microenvironment. By converting TGF-β from an immunosuppressant to an immunostimulant, these engineered CAR-T cells enhanced T-cell infiltration and reduced suppressive myeloid cells, improving survival in preclinical GBM models (Hou et al., 2024). CART with bispecific T-cell engager (CART.BiTE) cells were engineered to co-express an EGFRvIII-specific CAR and secrete EGFR-targeting BiTEs, enhancing the elimination of heterogeneous glioblastoma tumors. These cells recruited bystander T cells to target EGFR-positive tumor cells, overcoming the limitations of single-antigen CAR-T therapy. In mouse models, CART.BiTE effectively eradicated tumors without systemic BiTE circulation or toxicity against human skin grafts (Choi et al., 2019). Recently, a tri-modular CAR-T construct, CART-EGFR-IL13Rα2-dnTGFβRII has been developed to overcome the immunosuppressive GBM microenvironment by mitigating TGF-β-mediated suppression. This approach enhanced T-cell proliferation, functional responses, and bystander cell fitness while reducing TGF-β levels. *In vivo* studies confirmed its safety and efficacy in targeting GBM (Li N. et al., 2024).

### 3.3 CRISPR-based T-cell engineering in GBM immunotherapy

CRISPR-based genome editing is revolutionizing T-cell engineering for GBM immunotherapy by overcoming challenges like immune evasion and antigenic heterogeneity (Fang et al., 2024). CRISPR-Cas9 is used to knockout immune checkpoint genes (e.g., PD-1, TIGIT, LAG-3) in T cells, enhancing their anti-tumor activity by overcoming immunosuppressive effects in the GBM microenvironment (Li X. et al., 2024). Multiplexed CRISPR enables the creation of multi-targeted CAR-T cells that can target multiple antigens, such as EGFRvIII and IL13Rα2, and enhance persistence and resistance to immune evasion (Zhou et al., 2023). CRISPR is also used to engineer CAR-T cells to secrete cytokines like IL-12, reprogramming the tumor microenvironment for improved anti-tumor immunity. Additionally, CRISPR-based edits prevent T-cell exhaustion, maintaining long-lasting anti-tumor functionality. Finally, CRISPR helps develop CAR-T cells targeting both traditional and novel tumor-specific antigens, such as EGFRvIII, paving the way for personalized GBM immunotherapies (Martinez Bedoya et al., 2021; Nakazawa et al., 2020).

### 3.4 Macrophages secret bispecific T cell engagers (BiTEs)

Macrophages can be engineered to produce BiTEs, which are fusion proteins that link T cells to tumor cells, facilitating targeted cytotoxicity (Liu et al., 2025). Macrophages were genetically engineered to secrete a bispecific T cell engager (BiTE) targeting EGFRvIII in GBM, effectively activating T cells and reducing tumor burden in xenograft models. When co-expressing IL-12, macrophages further enhanced antitumor responses and prevented tumor growth. This approach harnesses macrophages’ natural tumor infiltration ability to improve local immunotherapy delivery and efficacy in GBM (Gardell et al., 2020).

## 4 Novel immune checkpoint inhibitors and their applications in GBM therapy

Recent advancements in immune checkpoint inhibition have expanded beyond PD-1 and CTLA-4 inhibitors, with promising candidates targeting additional immune checkpoints to overcome the immunosuppressive microenvironment in GBM. These emerging strategies aim to rejuvenate exhausted T cells and enhance anti-tumor immunity.

### 4.1 Lymphocyte-activation gene 3 (LAG-3) inhibitors

LAG-3 is a T-cell receptor that plays a significant role in immune regulation and exhaustion. Studies have shown that LAG-3 is upregulated on T cells within the TME of GBM, contributing to immune evasion and suppression of anti-tumor responses (Mair et al., 2021; Guo et al., 2024). Recent clinical trials have demonstrated that targeting LAG-3 with monoclonal antibodies, such as relatlimab, in combination with PD-1 inhibitors can enhance T-cell activation and promote a more robust anti-tumor immune response. The dual blockade of LAG-3 and PD-1 has shown synergistic effects in preclinical GBM models, leading to reduced tumor growth and prolonged survival (Harris-Bookman et al., 2018).

### 4.2 T-cell immunoglobulin and mucin-domain containing-3 (TIM-3) inhibitors

TIM-3 is another immune checkpoint receptor that plays a critical role in T-cell exhaustion. Overexpression of TIM-3 on T cells and myeloid cells in GBM has been linked to immune evasion (Hu W. et al., 2024; Ausejo-Mauleon et al., 2024). TIM-3 inhibitors are now being investigated to restore immune function and enhance tumor-targeting responses. Preclinical studies have suggested that targeting TIM-3 can reverse T-cell exhaustion, enhance CD8^+^ T-cell function, and increase tumor-infiltrating lymphocytes (TILs) in GBM. TIM-3 inhibition in syngeneic Diffuse intrinsic pontine glioma (DIPG) models extends survival and results in long-term disease-free survivors with immune memory. This antitumor effect is driven by direct TIM-3 inhibition in tumor cells, coordinated immune cell actions, and the secretion of chemokines/cytokines that promote a proinflammatory microenvironment, enhancing the antitumor immune response (Ausejo-Mauleon et al., 2023; Hu Y. et al., 2024; Lee and Lathia, 2023).

### 4.3 T-cell immunoreceptor with Ig and ITIM domains (TIGIT) inhibitors

TIGIT is an inhibitory receptor expressed on activated T cells and natural killer (NK) cells (Chauvin and Zarour, 2020). In GBM, TIGIT has been shown to suppress T-cell activation and promote immune tolerance in the TME. The TIGIT/CD155 axis is critical for glioblastoma’s immune evasion, yet targeting TIGIT alone has proven ineffective. Engineered synNotch-mediated activation of induced pluripotent stem cell-derived NK cells disrupts this axis by blocking CD73, preventing immunosuppressive adenosine buildup. This strategy shifts TIGIT/CD155 interactions towards activation, boosting NK cell cytotoxicity and achieving complete tumor eradication in glioblastoma models. By co-targeting TIGIT/CD155 and CD73, TME was reprogrammed, T cell recruitment was enhanced and M2 macrophages were reduced (Lupo et al., 2024). In addition, TIGIT expression was elevated on CD8^+^ and Tregs in the brain. Dual therapy with anti-PD-1 and anti-TIGIT significantly improved survival, enhancing effector T cell function and reducing suppressive Tregs and tumor-infiltrating dendritic cells (TIDCs) (Hung et al., 2018).

### 4.4 V-domain Ig suppressor of T cell activation (VISTA)

VISTA is a negative immune checkpoint that is highly expressed in GBM-associated myeloid cells and has been implicated in promoting immune suppression (Wang L. C. et al., 2022). Targeting VISTA has shown potential in preclinical models of GBM, where VISTA blockade enhanced T-cell responses and improved the efficacy of combination therapies with other immune checkpoint inhibitors (such as PD-1/PD-L1 inhibitors) (Ghosh et al., 2023; Petterson et al., 2023).

Beyond these, other immune checkpoints are emerging as potential therapeutic targets in GBM. B7-H3 (CD276), overexpressed on GBM cells and stromal components, inhibits T-cell activation and is associated with poor prognosis. Targeting B7-H3 with monoclonal antibodies or CAR-T therapy is currently under investigation (NCT04185038) (Vitanza et al., 2023). CD39 and CD73, two key ectonucleotidases involved in the adenosine pathway, contribute to an immunosuppressive TME by generating extracellular adenosine, which inhibits T-cell and NK-cell function. Inhibitors of these pathways are being explored in combination with immune checkpoint blockade (Takenaka et al., 2019; Goswami et al., 2020). Additionally, SIGLEC-15, a recently identified immunosuppressive molecule, functions similarly to PD-L1 in downregulating T-cell responses and represents a novel target in glioblastoma immunotherapy (Chen et al., 2023).

## 5 Emerging strategies to tackle immunotherapy resistance in GBM

GBM presents formidable challenges to treatment due to its inherent resistance mechanisms. Recent research has focused on innovative strategies to overcome these barriers, aiming to enhance therapeutic efficacy.

### 5.1 Combination of CAR-T therapies with immune checkpoint inhibitors improve the immunotherapy result

CAR-T therapy has shown promise in targeting GBM-specific antigens. However, its effectiveness is often limited by the immunosuppressive TME, which inhibits T-cell activity. To address this, combining CAR-T therapy with immune checkpoint inhibitors has been proposed. For instance, a study demonstrated that administering anti-PD-1 antibodies alongside CAR-T cells targeting EGFRvIII enhanced T-cell persistence and tumor regression in preclinical GBM models (NCT03726515) (Bagley et al., 2024a). This combination aims to counteract the TME’s suppressive effects, thereby improving therapeutic outcomes.

Siglec-9 has been identified as an immune checkpoint on macrophages in glioblastoma, where it limits T cell priming and the response to immunotherapy. Targeting Siglec-9 directly activates both CD4^+^ and CD8^+^ T cells by enhancing antigen presentation, chemokine secretion, and interactions with co-stimulatory factors (Mei et al., 2023). Furthermore, phosphoglycerate dehydrogenase (PHGDH) in endothelial cells (ECs) creates a hypoxic, immune-suppressive environment that helps GBM resist CAR-T therapy. Deleting PHGDH in endothelial cells reduces abnormal blood vessel growth, improves tumor oxygen levels, and increases T cell infiltration. Inhibiting PHGDH boosts T cell responses and makes GBM more responsive to CAR-T therapy. Targeting PHGDH could enhance T cell-based immunotherapy for GBM (Zhang D. et al., 2023).

Beyond CAR-T therapy, neoantigen-based vaccines and TCR-T therapy are being explored as complementary strategies to enhance immune responses in GBM. Neoantigen vaccines, designed to elicit tumor-specific T-cell activation, have been tested in combination with checkpoint blockade to sustain long-term immune surveillance (Keskin et al., 2019). Similarly, TCR-T cells, which recognize intracellular tumor-specific antigens presented by MHC molecules, offer a more precise targeting strategy compared to CAR-T cells. Ongoing clinical trials are evaluating whether these approaches can further improve anti-tumor immunity in GBM.

### 5.2 Novel approaches modulating the TME contributes to GBM immunotherapy

The TME in GBM is characterized by the presence of immunosuppressive cells, which contribute to therapeutic resistance. Strategies to modulate the TME include targeting these cell populations to restore anti-tumor immunity. For instance, inhibiting the colony-stimulating factor-1 receptor (CSF-1R) has been shown to deplete TAMs or reprogram them toward a pro-inflammatory phenotype, thereby enhancing the efficacy of immunotherapies (Quail et al., 2016). However, another study indicated that CSF-1R inhibition initially regresses tumors but leads to fibrosis-associated relapse in ∼50% of cases. Multi-omics analyses revealed that TGF-β-driven fibrosis creates pro-tumor niches, and targeting this pathway alongside CSF-1R inhibition improved survival in preclinical models (Watson et al., 2024).

Enhancing the persistence and infiltration of immune cells into the tumor is crucial for effective therapy. Genetic modifications of T cells to express chemokine receptors corresponding to ligands expressed by GBM improve their homing to the tumor site. CXCL11-armed oncolytic adenoviruses boost CAR-T cell efficacy and remodel the tumor microenvironment in glioblastoma (Wang G. et al., 2023). Moreover, engineering T cells to resist exhaustion by disrupting inhibitory pathways, such as the PD-1/PD-L1 axis, has been explored. For instance, CRISPR-Cas9-mediated knockout of PD-1 in CAR-T cells targeting IL13Rα2 resulted in increased T-cell persistence and anti-tumor activity in GBM models.

In addition, the immunosuppressive vascular niche in glioblastoma, driven by a mesenchymal-like endothelial cell population, promotes macrophage polarization and immunotherapy resistance through a Twist1/SATB1-mediated mechanism. Endothelial-derived osteopontin fosters immunosuppressive macrophage phenotypes, while Twist1 inhibition enhances T cell infiltration, reduces tumor growth, and improves CAR-T therapy efficacy (Yang et al., 2024). In addition, GBM harbors cancer-associated fibroblasts (CAFs) despite the absence of brain fibroblasts, as identified through single-cell transcriptomics and spatial analyses. CAFs interact with mesenchymal glioblastoma stem cells (GSCs) and M2 macrophages, promoting tumor growth via PDGF, TGF-β, osteopontin, and HGF signaling, making them a potential therapeutic target (Jain et al., 2023).

### 5.3 Modulation of cancer metabolism improves immunotherapy in GBM treatment

Targeting GBM’s metabolic reprogramming, including glycolysis, glutamine metabolism, and lipid oxidation, offers a promising strategy to overcome immunosuppression and enhance immunotherapy efficacy. By modulating these pathways, such as glycolysis inhibitors, glutaminase blockade, and fatty acid oxidation suppression improve T cell function and reshape the tumor microenvironment. One key metabolic target is lactate metabolism, which plays a crucial role in GBM-mediated immune suppression (Khan et al., 2024; Torrini et al., 2022). Tumor-derived lactate leads to the upregulation of ectonucleotidases CD39/CD73, increasing adenosine production and suppressing T cell activation (Sun et al., 2023). Additionally, lactylation of CCR8 in Tregs enhances their immunosuppressive function, further dampening anti-tumor immunity. Oxamate, a glycolysis inhibitor, has been shown to enhance CAR-T therapy efficacy by modulating tumor metabolism, specifically through the suppression of ectonucleotidases and inhibition of CCR8 lactylation. By disrupting lactate-driven immunosuppressive pathways, oxamate reduces the accumulation of adenosine and prevents lactylation-mediated regulatory T cell recruitment, thereby improving the anti-tumor activity of CAR-T cells (Sun et al., 2023). Beyond glycolysis, glutamine metabolism also plays a crucial role in immune suppression. Glutamine-derived α-ketoglutarate (α-KG) supports TAM polarization into an M2-like phenotype, which promotes tumor progression and inhibits T cell responses (Chung et al., 2020). Inhibiting glutaminase, the enzyme responsible for glutamine conversion, has been shown to enhance the efficacy of immune checkpoint blockade by shifting macrophages toward a pro-inflammatory M1 phenotype. Furthermore, lipid metabolism is emerging as another critical player in GBM immune evasion (Darwish et al., 2024). Fatty acid oxidation (FAO) is upregulated in Tregs and TAMs, supporting their immunosuppressive functions (Tamas et al., 2024). Pharmacological inhibition of FAO, such as using etomoxir, has demonstrated the potential to reprogram the TME, promoting CD8^+^ T cell activation and improving the efficacy of immunotherapy in preclinical GBM models (Darwish et al., 2024; Tanase et al., 2022). Collectively, these studies highlight the importance of metabolic modulation in GBM immunotherapy. By targeting key metabolic pathways, such as glycolysis, glutamine metabolism, and lipid oxidation, it is possible to reshape the immunosuppressive TME and enhance the effectiveness of CAR-T therapy, immune checkpoint inhibitors, and other immunotherapeutic strategies in GBM treatment (Caniglia et al., 2021).

### 5.4 Innovative delivery system overcomes the blood-brain barrier in GBM treatment

#### 5.4.1 Convection-enhanced delivery

The BBB remains a major challenge in delivering therapeutics to GBM. To overcome this, localized delivery methods such as convection-enhanced delivery (CED) and intrathecal administration have been explored (Vogelbaum and Aghi, 2015; D'Amico et al., 2021). CED enables direct infusion of therapeutic agents into the tumor or peritumoral brain tissue, bypassing the BBB and achieving higher local concentrations (Young and Aghi, 2022; Spinazzi et al., 2022). This approach has been used to enhance the delivery of CAR-T cells, checkpoint inhibitors, immunotoxins, and viral vectors encoding therapeutic genes (Shoji et al., 2016). Convection-enhanced locoregional delivery of nano-encapsulated genes generates ErbB2/Her2-specific CAR-macrophages for brainstem glioma immunotherapy (Gao et al., 2023).

#### 5.4.2 Nanoparticles and viral vectors increased BBB penetration

Nanoparticles and viral vectors are being investigated for BBB penetration, though these strategies are still in early development. Additionally, nanoparticles engineered to cross the BBB are being investigated as carriers for drugs, genes, or immune modulators, offering a promising approach to enhance delivery efficacy. Targeted mRNA nanoparticles alleviate blood-brain barrier disruption after ischemic stroke by regulating microglia polarization (Gao et al., 2024). A biomimetic nanodrug delivery platform, CpG-EXO/TGM, was developed to overcome glioblastoma treatment challenges by efficiently crossing the BBB and co-delivering chemotherapy and immune adjuvants. This system enhances drug accumulation in GBM cells, induces apoptosis, stimulates immune responses, and reduces postoperative recurrence, especially in combination with temozolomide (Cui et al., 2023). A lipid nanoparticle (LNP) platform with a dual-functional peptide (DAT-LNP) was developed to target glioma across the BBB/BBTB for immunotherapy. This system enables effective BBB penetration and brain accumulation post-intravenous administration while enhancing dendritic cell maturation, M1 macrophage polarization, and CD8^+^ T cell activation. By mitigating glioma’s immunosuppressive microenvironment, this approach elicits strong antitumor immunity (Tang et al., 2024).

#### 5.4.3 Focused ultrasound-mediated drug delivery

Focused ultrasound (FUS) is another promising strategy that temporarily disrupts the BBB in a controlled, non-invasive manner (Brighi et al., 2022). When combined with microbubbles, FUS creates transient openings in the BBB, allowing therapeutic agents to pass through and directly reach GBM tumors. This approach has been shown to enhance the delivery of chemotherapeutics, monoclonal antibodies, and gene therapy vectors (Berard et al., 2023). For example, FUS-mediated delivery of etoposide has demonstrated improved tumor response and survival in preclinical GBM models (Wei et al., 2021). In clinical trials, FUS combined with microbubbles has been successfully used to increase the delivery of immune checkpoint inhibitors to GBM, improving immune responses without causing significant systemic toxicity (Shen et al., 2022). This technique enhances the delivery of various therapeutics while minimizing adverse effects, making it a highly promising adjunct to existing therapies.

#### 5.4.4 BBB-disrupting agents

Pharmacological agents that transiently disrupt the BBB are also being explored as a means of facilitating drug delivery to GBM tumors (Wang J. et al., 2024). These agents work by targeting tight junction proteins that form the structural barrier of the BBB (Arms et al., 2024). For example, bradykinin receptor agonists, such as calcitonin gene-related peptide (CGRP), have been shown to enhance BBB permeability by modulating the endothelial tight junctions (Wang W. et al., 2022). This method increases the diffusion of therapeutic agents, including chemotherapeutics and immune modulators, into GBM tissue. Although these agents have shown promise in preclinical models, their use must be carefully regulated to prevent prolonged BBB disruption, which could lead to potential neurotoxicity. Nonetheless, the combination of BBB-disrupting agents with targeted therapies holds significant potential in overcoming one of the most critical barriers to effective GBM treatment.

Recent clinical trials have been initiated to optimize BBB modulation for immunotherapy. For instance, FUS-mediated BBB opening (NCT03712293) has shown potential in enhancing the delivery of CAR-T cells and checkpoint inhibitors to GBM tumors, improving immune infiltration (Park et al., 2021). Additionally, nanoparticle-based drug delivery strategies (NCT04221503) are being developed to transport immune-modulating agents across the BBB, increasing therapeutic efficacy while reducing off-target effects (Pal and Sheth, 2022). Engineered exosome-based delivery systems are also being investigated as a novel approach for transporting immune-stimulating molecules or CAR constructs directly into GBM sites. In summary, these emerging strategies aim to overcome the resistance mechanisms inherent in GBM by combining therapies, modulating the TME, engineering immune cells, and innovating delivery methods. Continued research in these areas holds the potential to significantly improve outcomes for patients with this challenging malignancy.

### 5.5 Personalized approaches in glioblastoma treatment

Personalized treatment strategies for GBM are increasingly focused on tailoring therapies to the unique genetic and molecular characteristics of individual tumors. One promising approach is the use of tumor mutational burden (TMB) and neoantigen-based vaccines (Yang et al., 2022). High TMB can be a predictor for better response to immune checkpoint inhibitors, and clinical trials are underway to evaluate the efficacy of personalized vaccines targeting neoantigens derived from patient-specific mutations (Xiong et al., 2024). These vaccines aim to stimulate the patient’s immune system to recognize and attack tumor cells more effectively. Additionally, single-cell RNA sequencing is being used to identify and map TME in detail, offering insights into the immune landscape and enabling the development of more tailored immunotherapies. For instance, combining checkpoint inhibitors with tumor-specific T-cell engagers (BiTEs) or bispecific antibodies is an innovative strategy that aims to enhance T-cell activation and infiltration into the GBM tumor while avoiding immune evasion mechanisms (Sternjak et al., 2021). The development of CD3 bispecific antibodies, which have shown potential in clinical trials for redirecting T-cells to target GBM tumor cells specifically (Gutova et al., 2024). Furthermore, liquid biopsy techniques are being explored to monitor dynamic changes in tumor genetics and immune responses, allowing for more adaptable treatment regimens based on the tumor’s evolution (Patel et al., 2024). These personalized therapies offer a more precise approach to combat GBM’s notorious resistance mechanisms and tumor heterogeneity, although further clinical validation is required to optimize their application.

## 6 Conclusion

In summary, while significant advancements have been made in the development of adoptive cell therapies for glioblastoma, several challenges persist. Tumor heterogeneity, the immunosuppressive microenvironment, and the physical barrier posed by the blood-brain barrier continue to impede therapeutic efficacy (Ravi et al., 2022). Nevertheless, innovative strategies, such as the optimization of CAR-T cell designs, modulation of cancer cell metabolism and TME, hold promise for overcoming these obstacles. Unlike immunotherapies that rely on immune system activation, small-molecule targeted therapies directly inhibit tumor-intrinsic pathways driving GBM progression and immune evasion. PI3K/AKT/mTOR inhibitors (e.g., everolimus, paxalisib) suppress tumor growth, while epigenetic modulators (EZH2, BET inhibitors) enhance immune recognition (Miklja et al., 2020). Metabolic inhibitors (IDH1/2, LDH inhibitors) reprogram tumor metabolism to reduce immune suppression. Importantly, small-molecule therapies and immunotherapy are not mutually exclusive but may complement each other. VEGF inhibitors (bevacizumab) can improve CAR-T therapy by reducing immunosuppressive myeloid cell infiltration and normalizing tumor vasculature, while TKIs (cabozantinib) enhance anti-tumor immune responses when combined with ICIs (Truffaux et al., 2015). The potential for immunotherapy and ACT to revolutionize GBM treatment is substantial, offering the possibility of durable responses and improved patient outcomes. With the rapid development of gene editing, biomaterials, and synthetic biology, novel strategies such as armored CAR-T cells, bispecific immune engagers, and locally implantable biomaterial scaffolds are emerging to enhance therapeutic efficacy. Moreover, advances in multi-omics profiling and artificial intelligence-driven drug discovery will facilitate the identification of optimal immunotherapy combinations, accelerating their clinical translation. Realizing this potential will require interdisciplinary collaboration among researchers, clinicians, bioengineers, and ethicists to address the multifaceted challenges inherent in developing and implementing these advanced therapies.

Combining immunotherapy with radiation, chemotherapy, or metabolic inhibitors can enhance anti-tumor efficacy and overcome resistance. For example, radiation therapy increases tumor immunogenicity and upregulates PD-L1 expression, potentially enhancing the efficacy of PD-1/PD-L1 inhibitors (NCT02667587) (Lim et al., 2022). Additionally, temozolomide (TMZ) induces immunogenic cell death, and its combination with immune checkpoint inhibitors may boost T-cell activation (NCT02658279) (Mellinghoff et al., 2022). In metabolic modulation, IDH1/2 inhibitors (e.g., ivosidenib) reduce immunosuppressive metabolites, helping restore T-cell function (NCT04056910). Optimizing these multimodal treatment strategies could significantly improve the effectiveness of immunotherapy in glioblastoma.

Here are four important pending questions in the glioblastoma cell therapy and immunotherapy field: (Weller et al., 2024): How can immune evasion mechanisms in the tumor microenvironment be overcome? Glioblastomas often develop mechanisms to evade immune detection, such as the recruitment of immunosuppressive cells (e.g., regulatory T cells) and the expression of immune checkpoint proteins. Understanding how to effectively modulate these pathways to enhance the effectiveness of immune-based therapies is crucial. (Schaff and Mellinghoff, 2023). What are the most effective strategies for overcoming the BBB in cell and gene therapies? The BBB limits the delivery and efficacy of therapeutic cells and molecules. Investigating novel methods to facilitate the safe and effective passage of engineered immune cells, such as CAR-T cells, across the BBB remains a major challenge. (Venkataramani et al., 2022). How can combination therapies (e.g., immune checkpoint inhibitors, targeted therapies, and cell-based therapies) be optimized to improve patient outcomes? Glioblastoma often becomes resistant to single-agent therapies. Identifying the optimal combinations and sequencing of treatments that can synergize to overcome resistance and provide long-term therapeutic benefits is a critical area of research (Liu et al., 2023a). What biomarkers can predict response to cell-based immunotherapies in glioblastoma? There is a need to identify reliable biomarkers to predict which patients will respond to therapies like CAR-T cells, CAR-NK cells, or other immunotherapies. These biomarkers would help personalize treatment and improve clinical outcomes by avoiding ineffective treatments. Answering these key questions is crucial for advancing glioblastoma cell therapy and immunotherapy. Overcoming immune evasion mechanisms will enhance immune response and improve therapy efficacy, while solving the challenge of crossing the blood-brain barrier will enable better delivery of therapies to the tumor site. Optimizing combination therapies will help overcome resistance and improve patient outcomes, and identifying predictive biomarkers will allow for more personalized treatment, reducing ineffective therapies and side effects. Together, addressing these questions will lead to more effective, personalized, and durable therapeutic strategies, significantly improving patient prognosis.

## Glossary

ACC: acetyl-CoA carboxylase
ACTs: Adoptive cell therapies
α-KG: α-ketoglutarate
BBB: blood-brain barrier
BiTEs: bispecific T cell engagers
CAFs: cancer-associated fibroblasts
CAR: chimeric antigen receptor
CAR-NK: CAR-natural killer cells
CART.BiTE: CART with bispecific T-cell engager
CED: convection-enhanced delivery
CGRP: calcitonin gene-related peptide
CNS: central nervous system
CSF-1R: stimulating factor-1 receptor
CTLA-4: cytotoxic T lymphocyte-associated antigen-4
DAT-LNP: dual-functional peptide
DCs: dendritic cells
DIPG: Diffuse intrinsic pontine glioma
ECs: endothelial cells
EGFRvIII: epidermal growth factor receptor variant III
FAO: Fatty acid oxidation
FUS: Focused ultrasound
GAMMs: glioma-associated microglia/macrophages
GBM: Glioblastoma
GSCs: glioblastoma stem cells
HIFs: hypoxia-inducible factors
ICB: immune checkpoint blockade
ICIs: Immune checkpoint inhibitors
IDO: indoleamine 2,3-dioxygenase
IL-12: interleukin-12
iNOS: inducible nitric oxide synthase
LGMN: legumain
MDSCs: myeloid-derived suppressor cells
MUC1-C: shorter C-terminal subunit
MHC: histocompatibility complex
PD-1: programmed death-1
PD-L1: programmed death ligand-1
PHGDH: phosphoglycerate dehydrogenase
ROS: reactive oxygen species
TAMs: tumor-associated macrophages
TGF-β: transforming growth factor-beta
TIDCs: tumor-infiltrating dendritic cells
TILs: tumor-infiltrating lymphocytes
TLS: tertiary lymphoid structure
TMB: tumor mutational burden
TME: tumor microenvironment
Tregs: regulatory T cells
